# Optimizing Ecological Restoration in Alpine Mining Areas Through Fertilization and Seeding-Rate Management: Insights from Vegetation–Soil Stoichiometry

**DOI:** 10.3390/plants15111640

**Published:** 2026-05-27

**Authors:** Nannan Hu, Xiaoyan Wang, Mingdan Song, Fuzhen Jiang, Kaibin Qi, Zhengpeng Li

**Affiliations:** 1 Academy of Animal Science and Veterinary, Qinghai University, Xining 810016, China; hu733n@163.com (N.H.); 18409481505@163.com (X.W.); 2 Academy of Agriculture and Forestry Sciences, Qinghai University, Xining 810016, China; hi0045@163.com (M.S.); qh815@163.com (F.J.); qikb@qhu.edu.cn (K.Q.)

**Keywords:** fertilization rate, seeding rate, nutrient stoichiometry, homeostasis, Muli mining area

## Abstract

The Muli mining area on the Qinghai–Tibet Plateau lies within a permafrost region where long-term coal mining has severely degraded native grassland ecosystems. To identify an effective restoration strategy, this study evaluated plant and soil ecological stoichiometry and stoichiometric homeostasis under different combinations of fertilization and seeding rates. A two-factor field experiment was conducted with three fertilization levels (F1–F3) and three seeding rates (S1–S3), using bare slag (BS) and natural grassland (NG) as reference controls. The F3S3 treatment produced the highest aboveground biomass (AGB), representing a 293.55% increase relative to NG. The F2S2 treatment significantly increased plant nitrogen (PN) and phosphorus (PP) contents. In addition, plant carbon-to-nitrogen (PC:PN), carbon-to-phosphorus (PC:PP), and nitrogen-to-phosphorus (PN:PP) ratios under the F2S2, F1S2, and F3S3 treatments, respectively, were closest to those of NG. The PN:PP ratio ranged from 6.05 to 8.20 (<14), indicating that plant growth in the restored plots remained primarily nitrogen-limited. Soil stoichiometric ratios (SOC:TN, SOC:TP, and TN:TP) under the F1S3, F1S1, and F1S2 treatments, respectively, were most similar to those of NG. Principal component analysis (PCA) showed that F3S3 produced the greatest short-term improvement in plant productivity and soil fertility, whereas F2S2 showed the most favorable stoichiometric homeostasis and C:N:P balance relative to natural grassland. Random forest modeling further identified soil total phosphorus, SOC:TN, and available phosphorus as the main factors controlling AGB formation. Overall, F3S3 is suitable for rapid short-term vegetation recovery, whereas F2S2 is more advantageous for long-term restoration when vegetation–soil stoichiometric balance and homeostatic stability are considered. Therefore, restoration projects in similar alpine permafrost mining areas should prioritize the F2S2 treatment to improve both ecological function and system stability.

## 1. Introduction

The Qinghai–Tibet Plateau, often referred to as the “Third Pole of the Earth” and the “Water Tower of Asia”, is highly sensitive to global climate change [1]. Alpine grasslands support rich plant diversity and account for 41.9% of China’s total grassland area. However, this fragile ecosystem has undergone widespread degradation in recent decades because of intensified human activities and climate warming, posing a serious threat to ecological security across the plateau [2]. The Muli mining area in Qinghai Province is located in the core region of the Qilian Mountains and forms part of the headwaters of the Datong River, a major tributary of the Yellow River [3]. Under the extreme climatic conditions of alpine mining areas, persistently low temperatures strongly inhibit soil microbial activity and slow the mineralization of soil organic matter. Frequent freeze–thaw cycles can also damage permafrost structure, release soil organic carbon, and alter water-migration pathways, thereby affecting soil quality and moisture dynamics [4]. In recent years, large-scale coal mining has destroyed surface vegetation and removed its soil-stabilizing and protective functions, further intensifying soil erosion, reducing grassland resources, and degrading mining-area grassland ecosystems [5].

Soil carbon (C), nitrogen (N), and phosphorus (P) are essential nutrients for plant growth, development, and reproduction [6]. In terrestrial ecosystems, these elements often act as primary limiting resources for vegetation and therefore play key roles in nutrient balance and biogeochemical cycling [7,8]. Their concentrations are important indicators of plant growth limitation and provide a critical link between soil nutrient status and plant physiological performance. Soil organic carbon (SOC), in particular, improves soil structural stability, water-holding capacity, and nutrient retention [9]. Soil N and P are widely recognized as major constraints on primary productivity, and their availability can influence grassland carbon sequestration [10]. In plant tissues, C, N, and P are fundamental structural components of proteins, nucleic acids, and other metabolites involved in physiological processes [11]. Accordingly, grassland vegetation growth is directly constrained by the supply and balance of soil C, N, and P; when these elements become imbalanced, normal vegetation development may be substantially inhibited.

Ecological stoichiometry examines the balance of energy and key elements, particularly C, N, and P, in biological systems [12]. It focuses on the contents, ratios, and ecological significance of these elements across different biological and environmental compartments, including plants and soils. Stoichiometric analysis has therefore become an important approach for revealing nutrient allocation patterns and diagnosing nutrient status in soil–plant systems. Soil ecological stoichiometry is used to clarify the coupled cycling of C, N, and P in soil, identify nutrient-limitation patterns, and assess their effects on ecosystem services [13]. Plant ecological stoichiometry characterizes elemental composition and ratios in plant tissues [14], providing a basis for evaluating plant nutrient limitation and community ecological strategies. By analyzing plant C:N:P stoichiometric patterns, researchers can assess plant nutrient status and adaptive responses to environmental change [15]. Previous studies have shown that plant elemental allocation is closely linked to soil nutrient stoichiometry [16]. Thus, examining the balance and allocation of C, N, and P in plant–soil systems is essential for understanding plant adaptation and feedback mechanisms under environmental change, especially in alpine ecosystems [17]. Under stresses such as soil nutrient limitation and climate change, plants may maintain relatively stable internal chemical composition through ecological stoichiometric homeostasis. This homeostatic regulation reflects plant adaptive potential and affects nutrient transformation, cycling, and biomass production at the ecosystem scale. Although ecological stoichiometric ratios have been widely studied, vegetation–soil stoichiometry and homeostasis during ecological restoration in the Muli mining area of the Qinghai–Tibet Plateau remain insufficiently understood. Addressing this gap can improve our understanding of nutrient-cycling mechanisms, identify key nutrient limitations, and provide theoretical guidance for vegetation restoration and ecosystem management in alpine mining environments.

Fertilization can improve soil physicochemical properties [18,19], soil enzyme activity [20], and microbial diversity [21], while also increasing grassland biomass. For example, Ba et al. [22] reported that the combined application of granular organic fertilizer and sheep manure in high-altitude mining areas did not significantly change grassland C content but significantly increased N and P contents and plant stoichiometric characteristics in artificially restored grassland. Yu et al. [23] found that a mixture of 50% sheep manure and 50% commercial organic fertilizer significantly increased soil total N, total P, organic matter content, and microbial community diversity. Yang et al. [21] further showed that increasing restoration intensity increased aboveground biomass and soil nutrients while significantly decreasing soil pH. In addition to fertilization, vegetation litter is an important source of soil nutrient input [24]. However, long-term coal mining in the Muli mining area has left vegetation cover extremely sparse and nutrient input severely limited. Researchers have therefore attempted vegetation restoration through supplementary seeding or reseeding. Lyu et al. [25] found that grass biomass in Qinghai grassland reached an optimum at a seeding rate of 9 g m^−2^. Previous studies have also shown that soil nutrients [26], C:N:P stoichiometric characteristics [27], and available nutrients [28] are significantly correlated with aboveground biomass. Importantly, soil nutrients regulate aboveground biomass not only directly, but also by influencing plant physiological nutrient processes. Plants may preferentially absorb nitrogen to promote growth, increasing plant N content, decreasing plant C:N ratios, and increasing N:P ratios, thereby facilitating aboveground biomass accumulation [29]. Conversely, phosphorus limitation can elevate plant C:P and N:P ratios and restrict vegetation growth. Given the severe ecological damage and nutrient-poor conditions caused by large-scale coal extraction in the Muli mining area of Qinghai [5], identifying appropriate restoration strategies is essential.

Against the background of climate-change-driven permafrost degradation, this study aimed to optimize fertilization and seeding-rate combinations to restore plant–soil stoichiometric balance and homeostasis, thereby enhancing the stability and climate resilience of alpine mining ecosystems. Based on a 2-year restoration monitoring experiment, we addressed the following questions from the perspective of ecological stoichiometry and stoichiometric homeostasis: (1) How do plant–soil C, N, and P stoichiometric characteristics and homeostasis respond to different restoration treatments? (2) Is plant growth during restoration primarily limited by nitrogen or phosphorus? (3) Which factors most strongly influence vegetation aboveground biomass during the early restoration stage? By answering these questions, this study seeks to clarify nutrient-balance mechanisms in soil–plant systems during mining-area ecological restoration and to provide a scientific basis for ecological restoration theory and practice in alpine mining areas.

## 2. Materials and Methods

### 2.1. Test Site

**Figure 1:**
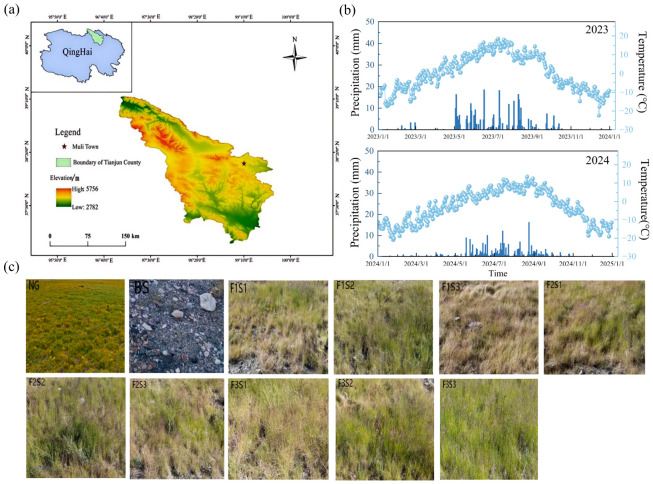
Overview of the experimental area. Shown are the location of the study area (**a**), distribution of rainfall and temperature (**b**), and experimental plot (**c**).

The experimental site was located at the former coal storage yard of the Juhugeng Mining Area in the Muli Coalfield, Qinghai Province, China, in the upper reaches of the Datong River (99°17′ E, 38°16′ N) (Figure 1). The site lies at an average elevation of approximately 4100 m and is characterized by a typical high-altitude continental climate with low temperatures and large diurnal temperature fluctuations. The maximum summer temperature reaches 19.8 °C, whereas winter temperature can fall to −34 °C, with a mean annual temperature of −1.68 °C. Mean annual precipitation is 516 mm, mean annual evaporation is 1190 mm, and precipitation is concentrated from May to September. The area is exposed to frequent winds throughout the year. The regional vegetation types are mainly alpine swamp and alpine meadow, with typical alpine characteristics, sparse vegetation, and a simple community structure. The forage growing season is short, lasting approximately 120 d. The soil is meadow soil, and soil moisture is mainly supplied by alpine snowmelt and atmospheric precipitation. Because of the high elevation and low temperature, the soil has a long freezing period; the presence of permafrost reduces soil permeability and causes seasonal waterlogging. Natural grassland vegetation is dominated by cold-tolerant, mesophytic, short-rhizomatous *Kobresia* species. The community exhibits cold-adapted characteristics, including low and dense clumps and prostrate growth, with no obvious vertical stratification. Dominant species include *Kobresia pygmaea*, *Kobresia capillifolia*, *Kobresia humilis*, and *Elymus nutans*. The native vegetation (undisturbed natural grassland in the area) has 70–90% coverage and a community height of 15–30 cm. Before the experiment, each independent plot was bare mine land without vegetation invasion. The initial soil physicochemical properties were as follows: total nitrogen (TN), 3.41 g kg^−1^; total phosphorus (TP), 0.77 g kg^−1^; total potassium (TK), 21.57 g kg^−1^; available nitrogen (AN), 20 mg kg^−1^; available phosphorus (AP), 9.7 mg kg^−1^; available potassium (AK), 191 mg kg^−1^; soil organic matter (SOM), 127.84 g kg^−1^; and pH, 9.56.

### 2.2. Experimental Design and Field Management

In alpine mining areas of Qinghai Province, large-scale restoration programs have commonly used high fertilization and high seeding rates [30]. Based on this practice, the present experiment examined whether fertilizer and seed inputs could be reduced and which combination would provide the best restoration outcome. A two-factor experimental design was therefore established to evaluate the effects of fertilization rate and seeding rate. Three fertilization levels were used: high fertilization (F3: sheep manure 495 m^3^ ha^−1^ + organic fertilizer 22.5 t ha^−1^ + forage special fertilizer 225 kg ha^−1^), medium fertilization (F2: sheep manure 330 m^3^ ha^−1^ + organic fertilizer 15 t ha^−1^ + forage special fertilizer 150 kg ha^−1^; two-thirds of the high fertilization rate), and low fertilization (F1: sheep manure 165 m^3^ ha^−1^ + organic fertilizer 7.5 t ha^−1^ + forage special fertilizer 75 kg ha^−1^; one-third of the high fertilization rate). Three seeding rates were also applied: high seeding rate (S3: 180 kg ha^−1^), medium seeding rate (S2: 120 kg ha^−1^; two-thirds of the high seeding rate), and low seeding rate (S1: 60 kg ha^−1^; one-third of the high seeding rate). The factorial combination of fertilization and seeding rates generated nine treatments (F1S1, F1S2, F1S3, F2S1, F2S2, F2S3, F3S1, F3S2, and F3S3), each with four replicates, giving 36 plots in total. Each plot was 24 m^2^ (4 m × 6 m), with a 1 m buffer between plots. The experiment followed a randomized complete block design. Bare slag (BS) and natural grassland (NG), both located within 500 m of the experimental plots, were used as reference controls.

The seed mixture consisted of four forage species: *Poa pratensis*, *Poa crymophila*, *Festuca sinensis*, and *Puccinellia tenuiflora*. The four species were mixed in equal proportions to maintain uniformity, enhance community stability, and avoid dominance by a single species. Seeds were mixed at a 1:1:1:1 ratio and sown on 24 June 2022. Before seeding, the surface layer of the coal-slag heap was mechanically crushed to produce substrate particles of <5 cm [30], followed by compaction and leveling. Fertilizers were applied according to the treatment design, and the mixed seeds were then broadcast evenly. After sowing, the plots were rotary-tilled, harrowed, and leveled, and a non-woven fabric cover was applied to reduce moisture loss and prevent wind erosion. Sheep manure was obtained locally, whereas the commercial organic fertilizer and forage special fertilizer were purchased from Qinghai Yipin Livestock Products Processing Co., Ltd. (Xining, China) (Table 1).

### 2.3. Soil and Plant Sampling

Plant sampling: Samples were collected in August 2023 and August 2024. In each restoration plot, one 1 m × 1 m quadrat was randomly established. At the same time, four 1 m × 1 m quadrats were randomly established in the natural grassland (NG) control area to characterize baseline vegetation under natural conditions. All aboveground vegetation within each quadrat was harvested to determine aboveground biomass (AGB). In total, 80 plant samples were collected over the 2 years.

Soil sampling: During the vegetation survey, soil samples were collected from the 0–10 cm layer in each quadrat using a soil auger and an S-shaped five-point sampling method. The five subsamples were mixed into one composite sample. After large stones and residual roots were removed, the samples were placed in self-sealing bags, transported to the laboratory, and divided into two portions. One portion was air-dried and passed through a 2 mm sieve for the determination of SOC, TN, and TP, whereas the fresh portion was passed through a 2 mm sieve for the determination of AN and AP. In total, 88 soil samples were collected over the 2 years.

### 2.4. Soil and Plant Nutrients Analysis

SOC was determined using the potassium dichromate external-heating oxidation method [31]. TN was measured using the semi-micro Kjeldahl method [31], and TP was determined by NaOH fusion followed by molybdenum–antimony colorimetry [31]. Soil available nitrogen (AN) was measured using the alkaline hydrolysis diffusion method, and soil available phosphorus (AP) was determined using sodium bicarbonate extraction followed by molybdenum–antimony colorimetric analysis [32]. After vegetation samples were collected from each quadrat, the fresh weight was recorded. The samples were then oven-dried at 105 °C for 1 h to stop metabolic activity and subsequently dried at 80 °C to constant weight. The dried material was used to determine plant carbon (PC), plant nitrogen (PN), and plant phosphorus (PP) contents. PC was measured using the potassium dichromate external-heating oxidation method, PN using the semi-micro Kjeldahl digestion method, and PP using NaOH fusion followed by molybdenum–antimony colorimetry.

Homeostasis reflects the capacity of a species to maintain a stable chemical composition under changing environmental conditions. The homeostasis index (H′) was calculated as follows [31]:

H′ = 1/m

where m is the slope of the log-transformed soil stoichiometric ratio regressed against the log-transformed plant stoichiometric ratio. According to Persson’s classification, a non-significant regression relationship (*p* > 0.05) indicates absolute homeostasis, meaning that plant stoichiometric composition is not detectably affected by variation in soil nutrient availability. When the regression relationship is significant (*p* < 0.05), homeostasis is classified according to the H′ value as follows: H′ < 1.33 indicates sensitivity; 1.33 < H′ < 2 indicates weak sensitivity; 2 < H′ < 4 indicates weak homeostasis; and H′ > 4 indicates homeostasis.

### 2.5. Statistical Analysis

Data were organized in Excel 2016, and statistical analyses were conducted using SPSS 22.0. Figures were generated using Origin 2021. All graphs were plotted using treatment means. Differences among treatments were assessed using Duncan’s multiple range test, with significance set at *p* < 0.05. A random forest model was constructed using the “randomForest” package in R 4.0.2, and Mantel tests were performed using the “linkET” package in R 4.0.2 to identify the main factors influencing vegetation aboveground biomass.

## 3. Results

### 3.1. Coupled Effects of Fertilization and Seeding Rates on Plant–Soil Relationships

Analysis of variance showed that, under the combined effects of fertilization and seeding rate, fertilization rate significantly affected most plant–soil C, N, and P contents and stoichiometric ratios, except PC and PN:PP (Table 2). Seeding rate significantly affected AGB, SOC, TN, TP, AN, AP, SOC:TP, and TN:TP. The interaction between fertilization rate and seeding rate significantly affected AGB, TN, TP, AN, AP, SOC:TN, SOC:TP, and TN:TP, indicating that the two factors jointly regulated key soil nutrient pools and stoichiometric relationships.

### 3.2. Interactive Effects of Fertilization and Seeding Rates on Aboveground Biomass

**Figure 2:**
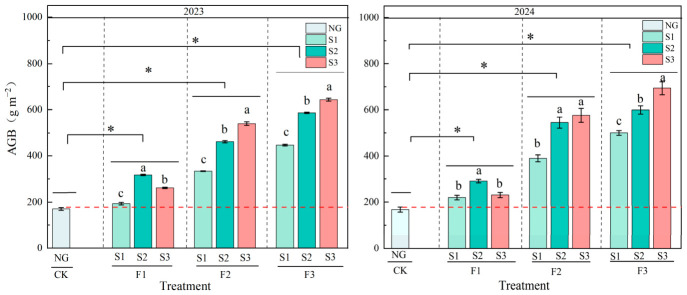
Effects of combined fertilization and seeding rates on aboveground biomass. Note: Lowercase letters indicate significant differences among seeding rates within the same fertilization level, whereas asterisks indicate significant differences between fertilization treatments and the control. AGB represents aboveground biomass.

Fertilization rate and seeding rate both had significant effects on aboveground biomass (AGB) (Table 2). Treatment effects on AGB were consistent across the 2 years, with the high-fertilization- and high-seeding-rate treatment producing the greatest increase. Compared with NG, this treatment increased AGB by 275.5% in 2023 and 311.9% in 2024 (*p* < 0.05). Relative to NG, all fertilization treatments significantly increased AGB (Figure 2). Averaged across the 2 years, F3, F2, and F1 increased AGB by 240.74%, 179.44%, and 48.58%, respectively, compared with NG (*p* < 0.05). Significant differences were also observed among seeding rates within the same fertilization level (Figure 2). Under low fertilization (F1), the medium seeding rate (S2) produced the highest 2-year AGB, with increases of 46.91% and 23.45% compared with S1 and S3, respectively (*p* < 0.05). Under medium (F2) and high (F3) fertilization, AGB was maximized under the high seeding rate (S3). Overall, F3S3 produced the highest AGB in both years, with a 293.55% increase relative to NG (*p* < 0.05). These results indicate a synergistic effect between high fertilization and high seeding rate, suggesting that their combination can overcome the limitations of either factor alone and maximize aboveground biomass.

### 3.3. Effects of Fertilization and Seeding Rate on Plant Carbon, Nitrogen, and Phosphorus Contents and Stoichiometric Characteristics

Fertilization rate significantly affected PN and PP contents but had no significant effect on PC content. In contrast, seeding rate did not significantly affect PC, PN, or PP contents (Table 2). Relative to NG, fertilization effects differed between the 2 years. Across the 2-year period, the increase in plant N and P contents was most pronounced under medium fertilization, whereas differences among seeding rates within the same fertilization level were not significant (Figure S1). In 2023, PC content ranged from 421.03 to 445.79. Compared with NG, fertilization significantly increased PC content, with increases of 5.30%, 5.17%, and 4.68% under F1, F3, and F2, respectively (*p* < 0.05). PN and PP contents ranged from 13.17 to 20.16 and from 1.82 to 2.53, respectively. F3 and F2 were significantly higher than F1 for PN, increasing PN by 33.8% and 29.99%, respectively (*p* < 0.05). F2 and F3 also significantly increased PP by 25.36% and 20.78%, respectively, compared with F1 (*p* < 0.05), although they did not differ significantly from NG. In 2024, PC content ranged from 411.32 to 431.15 and was significantly lower under fertilization than under NG. PN under F2 was significantly higher than under F1, increasing by 8.22% (*p* < 0.05), but remained 18.47% lower than NG (*p* < 0.05). PP did not differ significantly among fertilization treatments; however, compared with NG, F2 showed the largest increase, reaching 37.34% (*p* < 0.05). Across the 2 years, no significant differences were detected among seeding rates within the same fertilization level. Compared with NG, F2 significantly increased PP by an average of 24.80% (*p* < 0.05). Although PN decreased under all restoration treatments relative to NG, the F2 value (18.21) was closest to NG (19.44). These results indicate that moderate fertilization can enhance P uptake while avoiding excessive N accumulation, thereby supporting a more balanced nutrient-management strategy.

**Figure 3:**
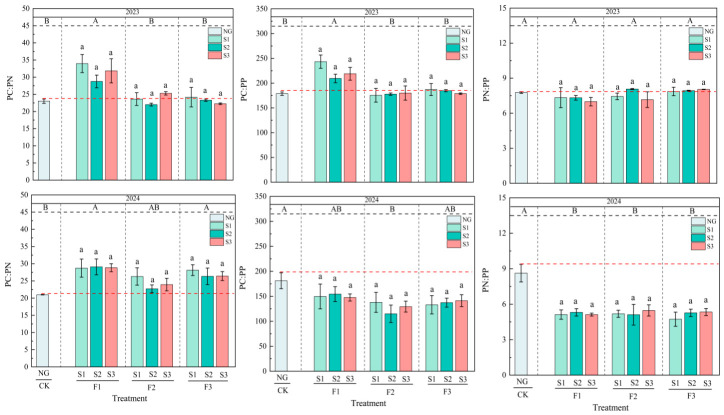
Effects of combined fertilization and seeding rates on plant C:N:P stoichiometric ratios. Note: Lowercase letters indicate significant differences among seeding rates within the same fertilization level, whereas uppercase letters indicate significant differences between fertilization treatments and the control. PC:PN, PC:PP, and PN:PP represent plant carbon-to-nitrogen, carbon-to-phosphorus, and nitrogen-to-phosphorus ratios, respectively.

Fertilization rate significantly affected plant C:N and C:P stoichiometry but had no significant effect on plant N:P stoichiometry. Seeding rate did not significantly affect plant C:N:P stoichiometry (Table 2). Over the 2 years, plant C, N, and P stoichiometric patterns were generally consistent, although differences among treatments were evident (Figure 3). On average, the PC:PN ratio under medium fertilization (F2; 23.97) was closest to the NG value (22.00). Within F2, the PC:PN ratio under the medium seeding rate (S2; 22.36) was also close to NG. The PC:PP ratio ranged from 146.36 to 196.50, and the 2-year mean PC:PP ratio under F1S2 (181.91) was closest to NG (179.95). The PN:PP ratio ranged from 6.05 to 8.20, and the 2-year mean PN:PP ratio under high fertilization (F3; 6.53) was closest to the NG value (8.20). Within F3, the PN:PP ratio under the high seeding rate (S3; 6.69) was closest to NG. These results indicate that plant C, N, and P stoichiometric responses were not synchronized. Therefore, fertilization strategies should be adjusted according to the target nutrient, while seeding rate can further fine-tune nutrient balance within a given fertilization level.

### 3.4. Effects of Fertilization and Seeding Rate on Soil Nutrients and C:N:P Stoichiometric Characteristics

Fertilization rate and seeding rate significantly affected SOC, TN, and TP contents (Table 2). Across the two experimental years, SOC, TN, and TP showed generally consistent treatment patterns: medium and high fertilization produced consistently higher contents than low fertilization, and differences were also observed among seeding rates within the same fertilization level (Figure S2). SOC content was significantly higher under F3, F2, and F1 than under BS, with 2-year mean increases of 78.10%, 61.82%, and 37.35%, respectively (*p* < 0.05). Compared with NG, SOC content was significantly lower under F1, F2, and F3, with 2-year mean decreases of 44.63%, 34.76%, and 28.20%, respectively (*p* < 0.05). Within the same fertilization level, seeding rate significantly affected SOC. Under F1, S3 and S2 were significantly higher than S1, with increases of 19.30% and 13.10%, respectively (*p* < 0.05). Under F2, S2 and S3 were significantly higher than S1, with increases of 19.38% and 15.40%, respectively (*p* < 0.05). Under F3, S3 produced the strongest increase, exceeding S1 and S2 by 21.12% and 6.68%, respectively (*p* < 0.05). Soil TN content ranged from 2.77 to 11.94. Compared with BS, F3, F2, and F1 significantly increased TN by 2-year means of 275.80%, 240.76%, and 125.27%, respectively (*p* < 0.05), whereas differences from NG were not significant. Seeding-rate differences were also observed within each fertilization level: S2 produced the greatest increase under F1, whereas S3 produced the greatest increase under F2 and F3. Soil TP content ranged from 0.34 to 1.66. Compared with BS, F3, F2, and F1 significantly increased TP by 2-year means of 343.93%, 282.48%, and 133.67%, respectively (*p* < 0.05). Compared with NG, F1 showed a slight but non-significant increase, whereas F3 and F2 significantly increased TP by 94.77% and 67.81%, respectively (*p* < 0.05). Within each fertilization level, S3 produced the greatest increase in TP. Under the combined effects of fertilization and seeding rate, F3S3 increased SOC by an average of 93.37% compared with BS over the 2 years (*p* < 0.05), although SOC remained lower than NG. F3S3 also produced the greatest increase in TN, followed by F3S2. Compared with BS, F3S3 and F2S3 increased TN by 331.05% and 269.31%, respectively (*p* < 0.05), and compared with NG, by 19.87% (*p* < 0.05) and 2.71%, respectively. F3S3 was also the most effective treatment for improving TP relative to both NG and BS. Overall, high fertilization combined with a high seeding rate markedly increased soil nutrient contents, likely because high seeding rates increased root biomass and organic residue inputs. Their coupling, therefore, represents an effective short-term strategy for rapidly improving soil fertility.

Fertilization rate and seeding rate significantly affected soil available nitrogen (AN) and available phosphorus (AP) (Table 2). Across the two experimental years, soil AN and AP increased progressively with fertilization rate. Under low and high fertilization, the high seeding rate produced the greatest improvement, whereas under medium fertilization, the medium seeding rate maximized AN, and the high seeding rate maximized AP (Figure S3). Compared with BS, all restoration treatments increased soil AN and AP. Compared with NG, soil AP was significantly higher under all restoration treatments. Soil AN was significantly higher under all fertilization treatments than under BS, with F3 producing the strongest increase. Within F3, S3 increased soil AN by 21.81% and 77.71% compared with S2 and S1, respectively (*p* < 0.05). Compared with both NG and BS, all fertilization treatments significantly increased soil AP, with F3 producing the largest improvement. Within the same fertilization level, S3 consistently produced the highest soil AP content. Overall, F3S3 produced the greatest improvement in both AN and AP. These results indicate that high fertilization coupled with a high seeding rate can substantially improve the supply capacity of available soil nutrients and rapidly activate soil fertility.

**Figure 4:**
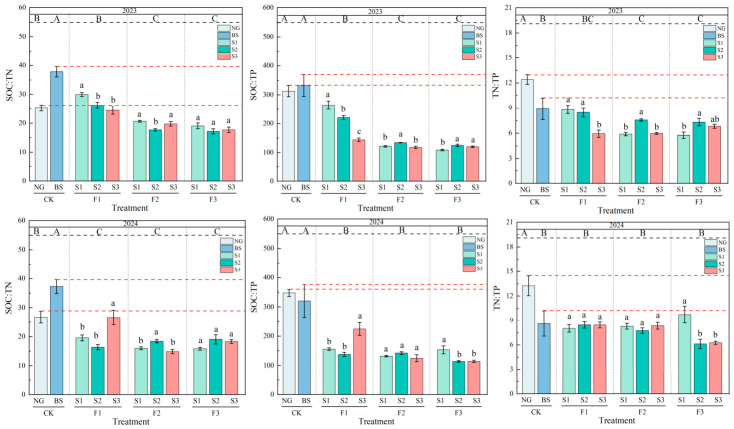
Interactive effects of fertilization rate and seeding rate on soil C, N, and P stoichiometric characteristics. Note: Lowercase letters indicate significant differences among seeding rates within the same fertilization level, whereas uppercase letters indicate significant differences between fertilization treatments and the control. SOC:TN, SOC:TP, and TN:TP represent soil carbon-to-nitrogen, carbon-to-phosphorus, and nitrogen-to-phosphorus ratios, respectively.

Fertilization rate significantly affected SOC:TN, SOC:TP, and TN:TP ratios, whereas seeding rate significantly affected SOC:TP and TN:TP but not SOC:TN (Table 2). Across the 2 years, soil stoichiometric ratios generally declined relative to NG and BS, with the strongest decreases occurring under high fertilization (Figure 4). Compared with BS, F3, F2, and F1 reduced SOC:TN by 2-year means of 52.45%, 52.38%, and 36.45%, respectively (*p* < 0.05). Compared with NG, F3 and F2 reduced SOC:TN by 31.30% and 31.20%, respectively (*p* < 0.05). Within F1, S3 was significantly higher than S2, with a 2-year mean increase of 19.91% (*p* < 0.05). SOC:TP decreased significantly under all fertilization treatments relative to both NG and BS. Within F2, S2 increased SOC:TP by 13.97% and 9.01% compared with S3 and S1, respectively (*p* < 0.05). Soil TN:TP also decreased significantly under all fertilization treatments relative to NG and BS. Compared with NG, F3, F2, and F1 reduced TN:TP by 2-year means of 45.42%, 43.02%, and 37.37%, respectively (*p* < 0.05), and by an average of 20.21% relative to BS (*p* < 0.05). Within F1, S2 and S1 increased TN:TP by 17.75% and 17.22%, respectively, compared with S3 (*p* < 0.05). Overall, under combined fertilization and seeding treatments, the SOC:TN ratio under F1S3 (25.55) was closest to NG (26.00). The SOC:TP ratio was closest to NG under F1S1, whereas the TN:TP ratio was closest to NG under F1S2. These results indicate that low fertilization is more conducive to maintaining soil C:N:P stoichiometric balance, while seeding-rate adjustment can further optimize nutrient ratios.

### 3.5. Vegetation–Soil PCA Analysis

**Figure 5:**
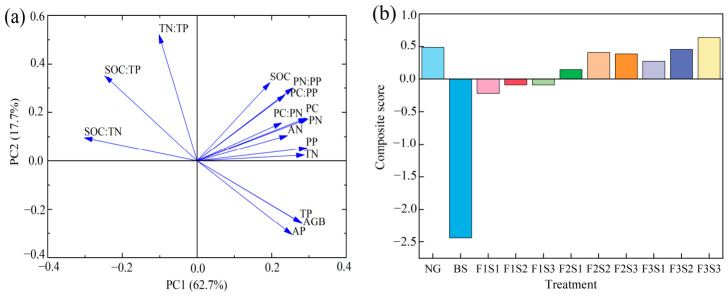
Vegetation–soil PCA analysis. Note: Figure (**a**) represents the principal component analysis, and Figure (**b**) represents the comprehensive scores of each treatment.

Principal component analysis (PCA) of vegetation and soil variables showed that the first principal component (PC1) explained 62.7% of the total variance and the second principal component (PC2) explained 17.7%. Together, these two components explained 80.4% of the cumulative variance (Figure 5a), indicating that they captured most of the variation in the dataset. Composite PCA scores showed that F3S3 ranked highest among all restoration treatments, followed by F3S2 and F2S2 (Figure 5b), suggesting that high fertilization combined with a high seeding rate most strongly enhanced vegetation productivity and soil fertility in the short term.

### 3.6. Plant–Soil Stoichiometric Homeostasis (H′) and Factors Influencing Aboveground Biomass

The homeostasis analysis of plant and soil stoichiometric ratios (Figure S4) showed absolute homeostasis between plant PC:PN and soil SOC:TN. Plant PC:PP and soil SOC:TP exhibited sensitive states under F1S1 (H′ = 1.05, H′ < 1.33) and F2S1 (H′ = 0.30, H′ < 1.33), indicating that plant PC:PP was strongly driven by soil SOC:TP and that plant nutrient-regulation capacity was limited under these treatments. Under F1S3, plant PC:PP and soil SOC:TP exhibited a weakly sensitive state (H′ = 1.37, 1.33 < H′ < 2), suggesting some buffering capacity. Plant PN:PP and soil TN:TP showed a weakly sensitive state under F1S3 (H′ = 1.42, 1.33 < H′ < 2) and sensitive states under F2S1 (H′ = 0.97, H′ < 1.33) and F2S3 (H′ = 1.14, H′ < 1.33), indicating that plant PN:PP was susceptible to variation in soil TN:TP.

**Figure 6:**
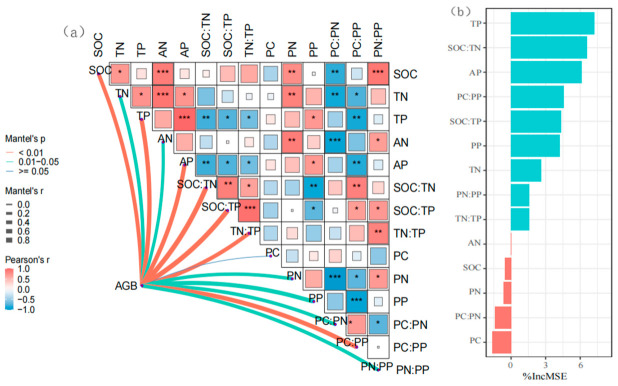
Analysis of drivers of aboveground biomass. Note: (**a**) shows the Mantel test. AGB denotes aboveground biomass; SOC, TN, and TP represent soil organic carbon, total nitrogen, and total phosphorus, respectively; AN and AP denote available nitrogen and available phosphorus, respectively; SOC:TN, SOC:TP, and TN:TP denote soil carbon-to-nitrogen, carbon-to-phosphorus, and nitrogen-to-phosphorus ratios, respectively; PC, PN, and PP represent plant carbon, nitrogen, and phosphorus, respectively; and PC:PN, PC:PP, and PN:PP denote plant carbon-to-nitrogen, carbon-to-phosphorus, and nitrogen-to-phosphorus ratios, respectively. Line thickness represents the strength of the relationships between AGB and vegetation–soil nutrients and stoichiometric ratios. *, **, and *** indicate significant correlations at *p* < 0.05, *p* < 0.01, and *p* < 0.001, respectively; (**b**) shows the random forest model, which quantifies variable importance for AGB using percentage increase in mean squared error (%IncMSE). Higher %IncMSE values indicate greater predictive contribution. The green bars represent the positive contribution to aboveground biomass, and the red bars represent the negative contribution to aboveground biomass.

The Mantel test (Figure 6a) showed significant correlations between aboveground biomass (AGB) and multiple plant–soil indicators, including SOC, TN, TP, AN, AP, SOC:TN, SOC:TP, TN:TP, PN, PP, PC:PN, PC:PP, and PN:PP. Plant PN was significantly positively correlated with soil SOC, TN, and AN, whereas plant PC:PN was significantly negatively correlated with soil SOC, TN, and AN. Plant PC:PP was significantly negatively correlated with soil TP and AP but significantly positively correlated with SOC:TN and SOC:TP. Plant PN:PP was significantly positively correlated with soil SOC, AN, SOC:TP, and TN:TP. These relationships indicate that plant internal stoichiometric balance was strongly regulated by soil nutrient availability. In general, plant nutrient contents (PN and PP) were positively associated with corresponding soil nutrient contents, whereas plant stoichiometric ratios (PC:PP and PN:PP) covaried with soil stoichiometric ratios. This pattern suggests close coupling between plant tissue elemental balance and soil nutrient-supply patterns in this restoration system. To further identify the key drivers of AGB and their relative contributions, a random forest model was constructed (Figure 6b). The model identified soil TP, SOC:TN, and AP as the main factors influencing AGB, each with relatively high contribution rates.

## 4. Discussion

### 4.1. Effects of Fertilization Rate and Seeding Rate on Vegetation Biomass and Plant Nutrient Stoichiometry

In this study, AGB increased with fertilization rate, and under medium to high fertilization, biomass was maximized at the high seeding rate. These findings are consistent with previous studies. A key distinction of the present study is that the effect of seeding rate became evident only when nutrient supply was sufficient, indicating that nutrient availability, rather than plant density alone, was the dominant limiting factor in the extremely infertile soils of alpine mining areas. This result suggests that restoration in similar mining areas should first alleviate nutrient limitation through fertilization before the potential benefits of high seeding rates can be fully realized. Plant nutrient uptake is closely linked to biomass formation [33]. Plants can maintain relatively stable nutrient contents through physiological regulation even when external nutrient availability changes. In the present study, medium fertilization most effectively increased PC, PN, and PP contents relative to NG. This may be because medium fertilization supplied an appropriate amount of limiting nutrients, especially nitrogen, thereby alleviating nutrient deficiency in the alpine mining substrate while avoiding the nutrient imbalance associated with excessive fertilization. Moderate nutrient supply may also promote coordinated aboveground and belowground growth, enhance root nutrient acquisition, and increase plant C, N, and P accumulation. In addition, medium fertilization may better maintain rhizosphere microbial activity and function, promoting nutrient cycling and availability and indirectly improving plant nutrient-uptake efficiency [34]. According to the growth-rate hypothesis, rapidly growing plants generally exhibit lower C:N and C:P ratios, reflecting greater allocation of nutrients to metabolic processes rather than structural biomass [35]. The plant N:P ratio is commonly used to diagnose nutrient limitation: N:P > 16 indicates P limitation, N:P < 14 indicates N limitation, and values between 14 and 16 suggest N–P co-limitation [36]. In this study, PN:PP ranged from 6.05 to 8.20 (<14), indicating that plant growth in the Muli mining area was primarily N-limited, consistent with the widespread N limitation reported for alpine regions [37]. Furthermore, PC:PN, PC:PP, and PN:PP ratios were closest to NG under F2S2, F1S2, and F3S3, respectively. Medium fertilization likely provided sufficient N for plant growth [38], while the medium seeding rate balanced interspecific competition and nutrient supply, reducing excessive resource competition among individuals, consistent with Cheng et al. [39]. Under low fertilization, soil P supply was limited; a medium seeding rate may have reduced P competition compared with high seeding density while improving P acquisition relative to low seeding density, making PC:PP closer to the low-P adaptive status of NG. Under high fertilization, sufficient N and P were supplied to the soil [40]. At high seeding density, planted grasses may have acquired both N and P more effectively, resulting in a plant N:P ratio closer to NG, a pattern supported by Tao et al. [41]. These findings suggest that medium fertilization combined with moderate seeding density is the most favorable approach for restoring stoichiometric balance in this mining area. More broadly, shifting plant stoichiometric homeostasis toward natural-grassland conditions requires the coordinated optimization of fertilization rate and seeding density [42]. Thus, nutrient addition alone, without density regulation, is unlikely to fully restore nutrient balance to the natural-grassland state. Whereas previous studies have often examined fertilization [43,44] or seeding rate [45] separately, this study provides direct evidence that their interaction is critical for stoichiometric restoration.

### 4.2. Effects of Fertilization and Seeding Rate on Soil Nutrients and Soil Stoichiometric Ratios

Soil C, N, and P contents are important indicators of mine-restoration effectiveness [46], and improving these nutrient pools is central to ecological restoration in the Muli mining area. In this study, SOC, TN, and TP showed generally consistent 2-year trends across treatments, with medium and high fertilization producing higher values than low fertilization. This pattern is consistent with the view that exogenous C, N, and P inputs stimulate microbial activity and accelerate nutrient cycling, as reported by Chu et al. [47] and Shi et al. [38]. The present study further showed that soil nutrient increases were greatest under high fertilization combined with a high seeding rate. This may be because dense root networks under high seeding density reduced nitrate leaching, while root exudates mobilized part of the fixed phosphorus pool, jointly promoting AN and AP accumulation [48,49]. Although fertilization increased soil nutrient pools in the short term, SOC and AN remained lower than those in natural grassland. This likely reflects the loose soil structure of alpine mining substrates, which can promote nitrate loss from the root zone under extreme snowfall or rainfall events [50]. At the same time, newly restored vegetation may still have insufficient biomass and root capacity to intercept and absorb all available nitrogen, further increasing the risk of N loss. Lower SOC relative to natural grassland likely reflects the limited litter return in newly restored vegetation despite fertilization-induced biomass increases. In addition, under high fertilization, N and P not retained by plants or soil, particularly mobile nitrate and soluble P, may migrate to surrounding water bodies under sparse vegetation cover, intense freeze–thaw cycles, and precipitation-driven leaching, creating a potential risk of non-point-source pollution. Soil stoichiometric ratios further revealed nutrient-limitation status. In this study, the 2-year mean soil C:N ratio under restoration treatments ranged from approximately 17.29 to 25.55, higher than the reported averages for China (12.3) and the world (11.8) [51]. The soil N:P ratio ranged from 6.53 to 12.82, with all values below 14. Based on the threshold criteria that SOC:TN < 30 and TN:TP < 14 indicate N limitation, nitrogen appears to be the main factor limiting vegetation growth in this region [52,53]. This likely reflects a relatively sufficient SOC pool but comparatively low N availability, which constrains plant N acquisition. In the severely disturbed alpine mining ecosystem, soil N can be depleted through erosion and mineralization losses, whereas P is relatively more stable, making N the most critical limiting element. Furthermore, plants acquire N mainly through root uptake of soil N, a pathway with limited flexibility [54]. The SOC:TN ratio under F1S3 (25.55) was close to NG (26.00), the SOC:TP ratio was closest to NG under F1S1, and the TN:TP ratio was closest to NG under F1S2. These patterns may be associated with insufficient N and P input under low fertilization: plants absorb some N and P during growth, while organic matter decomposition remains inhibited [55]. Differences in soil stoichiometry among seeding rates are likely related to increased C inputs from root exudates and litter under higher seeding density. However, because N uptake does not increase synchronously under limited fertilization, SOC:TN remains relatively high. Under moderate seeding rates, plants can avoid both high-density competition and the nutrient acquisition constraints associated with very low plant density, resulting in a more natural TN:TP ratio. Overall, the interaction between fertilization and seeding density regulates not only the absolute size of soil nutrient pools but also their stoichiometric balance. This study highlights the importance of coordinated nutrient and density management for soil nutrient restoration and stoichiometric convergence, and it indicates that fertilization without density regulation may constrain soil restoration and increase environmental risk.

### 4.3. Plant–Soil Stoichiometric Homeostasis (H′) and Factors Influencing Aboveground Biomass

Ecological stoichiometry describes the balance of multiple chemical elements in ecosystems and the ecological processes that regulate that balance [56]. Stoichiometric homeostasis refers to the capacity of organisms to maintain relatively stable internal elemental composition despite environmental variation [57]. Nutrient allocation among plant organs can alter relationships between soil C, N, and P contents and their stoichiometric ratios [58], while plants can maintain relatively stable internal nutrient composition through self-regulation under changing soil nutrient conditions. In this study, plant PC:PN and soil SOC:TN showed strict homeostasis, suggesting that plant N uptake remained relatively stable across soil nutrient conditions. In contrast, plant PC:PP and soil SOC:TP showed a sensitive state under F1S1 and a weakly sensitive state under F1S3. Under low fertilization, insufficient exogenous nutrient input likely reduced soil P availability, forcing plants to adjust internal C:P ratios to improve P-use efficiency and producing a sensitive response. Under F1S3, the high seeding rate may have intensified competition for limited P, prompting plants to enhance P uptake and redistribution. This could partially buffer disturbances to internal C:P stoichiometry and explain the weakly sensitive response. Because plant P acquisition under low fertilization depends mainly on root activation and uptake of soil P, root overlap and competition under high seeding density may stimulate root exudation and P-acquisition capacity, thereby improving homeostatic regulation compared with F1S1. Plant PN:PP and soil TN:TP showed a weakly sensitive state under F1S3, sensitive states under F2S1 and F2S3, and absolute homeostasis under the other treatments. This pattern may be explained by two mechanisms. First, under low fertilization with high seeding rate (F1S3) and medium fertilization with low or high seeding rate (F2S1 and F2S3), the match between nutrient input and plant density was imbalanced, causing the soil N:P supply ratio to deviate from the optimum range for plant uptake and inducing nutrient-structure imbalance. Second, high seeding density intensified competition among individuals for nutrient resources. When the soil N:P supply ratio was imbalanced, this competition further amplified nutrient limitation and weakened the capacity of plants to maintain internal stoichiometric balance. In contrast, when nutrient supply and seeding density were better coordinated, plants maintained more stable internal N:P ratios through regulation of nutrient uptake, allocation, and physiological turnover, thereby showing stronger homeostatic characteristics [59]. These results indicate that coordinated optimization of fertilization and seeding density is essential for maintaining plant nutrient homeostasis and shifting restored communities toward the natural-grassland state.

Mantel test analysis showed significant correlations between AGB and multiple vegetation and soil elemental indicators. The correlation with plant C content was weak, probably because C concentration in plant tissues is relatively stable and is not a primary driver of AGB formation. Although AGB is ultimately derived from the allocation of photosynthetic products, this process is strongly regulated by N and P supply, explaining the significant relationships between AGB and vegetation–soil N and P nutrients and stoichiometric ratios. Correlation analysis further showed that plant PN was significantly positively correlated with soil TN, while plant PP was significantly positively correlated with soil TP and AP but negatively correlated with SOC:TP. Plant PC:PP was significantly positively correlated with SOC:TP but negatively correlated with soil TP. These results indicate that plant internal stoichiometric balance is regulated by soil nutrient availability and that, under fertilization, increased soil C, N, and P contents promote plant nutrient uptake and nutrient accumulation [60]. The random forest model identified soil TP, SOC:TN, and AP as the most important factors influencing AGB. This result can be interpreted in two ways. First, phosphorus is a key nutrient for plant growth, and both total and available P showed high contribution rates, indicating that P supply and its effective forms strongly regulate AGB in this grassland restoration system. Second, the high contribution of SOC:TN suggests that the soil C:N balance influences microbial activity and N transformation, thereby affecting plant N acquisition. The interaction between N availability, which controls plant growth rate, and P uptake and utilization ultimately determines AGB accumulation [61]. It is worth noting that, in this study, the random forest analysis showed a higher contribution of soil phosphorus to aboveground biomass than nitrogen-related factors, which does not conflict with the conclusion of nitrogen limitation inferred from the plant N:P ratio. Random forest analysis reflects the statistical contribution of each factor to variation in aboveground biomass, which is influenced by factors such as spatial heterogeneity and thus cannot be directly equated with actual nutrient limitation intensity. The plant N:P ratio directly reflects the internal nutrient balance of plants and is a classic ecological indicator for assessing nutrient limitation [36]. In this study, the N:P ratios in all plots were consistently below 14 [37]. Based on these plant physiological characteristics, we conclude that nitrogen is the primary limiting factor for plant growth in the Muli mining area.

## 5. Conclusions

This study used a two-factor field experiment combining fertilization and seeding rates, with bare slag and natural grassland as reference controls, to evaluate treatment effects on biomass, plant–soil nutrients, stoichiometric ratios, and stoichiometric homeostasis. The F3S3 treatment produced the greatest aboveground biomass and therefore showed a clear advantage for rapid short-term vegetation recovery. However, high-input restoration increased soil N and P while SOC remained lower than that of natural grassland, indicating that biomass recovery alone does not necessarily represent stoichiometric recovery. Plant N:P ratios remained below 14 across all treatments, demonstrating persistent nitrogen limitation. Homeostasis analysis showed stable C:N coupling, whereas C:P and N:P relationships were more sensitive to treatment conditions. Overall, the F2S2 treatment provided the most favorable balance between nutrient supply, plant density, and stoichiometric stability. These findings indicate that ecological restoration in degraded alpine mining areas should prioritize the reconstruction of vegetation–soil nutrient balance rather than simply maximizing biomass or rapidly reproducing the visual appearance of natural grassland. Moderate nutrient input combined with moderate plant density offers a more sustainable and replicable pathway for restoring ecosystem nutrient function in similar degraded alpine environments.

This study has several limitations. First, the experiment was based on only 2 years of restoration data, and the long-term stability of the observed restoration effects remains uncertain. Second, although the study clarified the broad effects of fertilization and seeding-rate treatments on vegetation–soil C, N, and P contents and stoichiometric characteristics in an alpine mining area, the homeostasis indices were calculated from a limited set of treatment gradients. Whether these conclusions can be generalized to broader and more continuous natural environmental gradients requires further verification. Third, the functional responses of microbial communities to the restoration measures remain unclear. Future research should therefore validate the stoichiometric homeostasis patterns identified here across wider natural gradients and should integrate measurements of soluble soil nutrients, microbial activity, and high-throughput sequencing. Such work would help clarify how soil nutrient availability and microbial functional responses under different restoration treatments regulate vegetation recovery and would provide a mechanistic basis for ecological restoration in alpine mining areas.

## Figures and Tables

**Table 1:** Basic physical and chemical properties of selected fertilizers.

Type	Organic Matter (SOM) (%)	Total Nitrogen (TN) (%)	Total Phosphorus (TP) (%)	Total Potassium (TK) (%)	Soil pH	Moisture Content (%)
Sheep manure	41.43	1.168	0.738	1.396	7.86	35.08
Commercial organic fertilizer	31.74	1.044	0.715	1.657	7.76	28.82
Forage special fertilizer	—	18	14	8	—	—

**Table 2:** ANOVA results for the interactive effects of fertilization rate and seeding rate on plant–soil C, N, and P contents and stoichiometric ratios.

Source of Variation		AGB	PC	PN	PP	PC:PN	PC:PP	PN:PP	SOC	TN	TP	AN	AP	SOC:TN	SOC:TP	TN:TP
Fertilization rate	F	882.02	0.05	18.09	6.81	17.19	10.99	0.87	87.01	135.65	230.37	892.99	1340.46	66.61	123.36	8.6
	*p*	***	ns	***	**	***	***	ns	***	***	***	***	***	***	***	**
Seeding rate	F	186.99	0.35	1.55	0.16	1.75	0.54	0.41	41.91	31.67	47.39	221.42	269.08	2.09	5.11	5.53
	*p*	***	ns	ns	ns	ns	ns	ns	***	***	***	***	***	ns	**	**
Fertilization rate × Seeding rate	F	24.19	1.72	0.27	0.33	0.26	0.26	0.25	1.84	5	3.27	58.42	22.84	4.04	3.19	2.62
	*p*	***	ns	ns	ns	ns	ns	ns	ns	**	*	***	***	**	*	*

Note: *, **, and *** indicate significance at *p* < 0.05, *p* < 0.01, and *p* < 0.001, respectively; ns indicates no significant effect. AGB represents aboveground biomass. PC, PN, and PP represent plant carbon, nitrogen, and phosphorus contents, respectively. PC:PN, PC:PP, and PN:PP represent plant carbon-to-nitrogen, carbon-to-phosphorus, and nitrogen-to-phosphorus ratios, respectively. SOC, TN, and TP represent soil organic carbon, total nitrogen, and total phosphorus contents, respectively. AN and AP represent available nitrogen and available phosphorus, respectively. SOC:TN, SOC:TP, and TN:TP represent soil carbon-to-nitrogen, carbon-to-phosphorus, and nitrogen-to-phosphorus ratios, respectively.

## Data Availability

The data presented in this study can be requested from the corresponding author.

## Supplementary Materials

The following supporting information can be downloaded at: https://www.mdpi.com/article/10.3390/plants15111640/s1, Figure S1. Effects of fertilizer application rate and seeding rate on plant carbon, nitrogen, and phosphorus contents. Figure S2. Effects of fertilization and seeding rate on soil carbon, nitrogen, and phosphorus contents. Figure S3. Effects of Fertilizer and Seeding Rates on Soil Available Nutrients. Figure S4. Analysis of the plant-soil stoichiometric homeostasis index.
