# Supplementary material for: Optimizing Ecological Restoration in Alpine Mining Areas Through Fertilization and Seeding-Rate Management: Insights from Vegetation–Soil Stoichiometry
Source: Plants (Basel). 2026 May 27;15(11):1640. doi: 10.3390/plants15111640 (PMC13259500; doi:10.3390/plants15111640)
Supplement: Supplementary file 1 [file plants-15-01640-s001.zip › plants-4276488-supplementary.pdf]

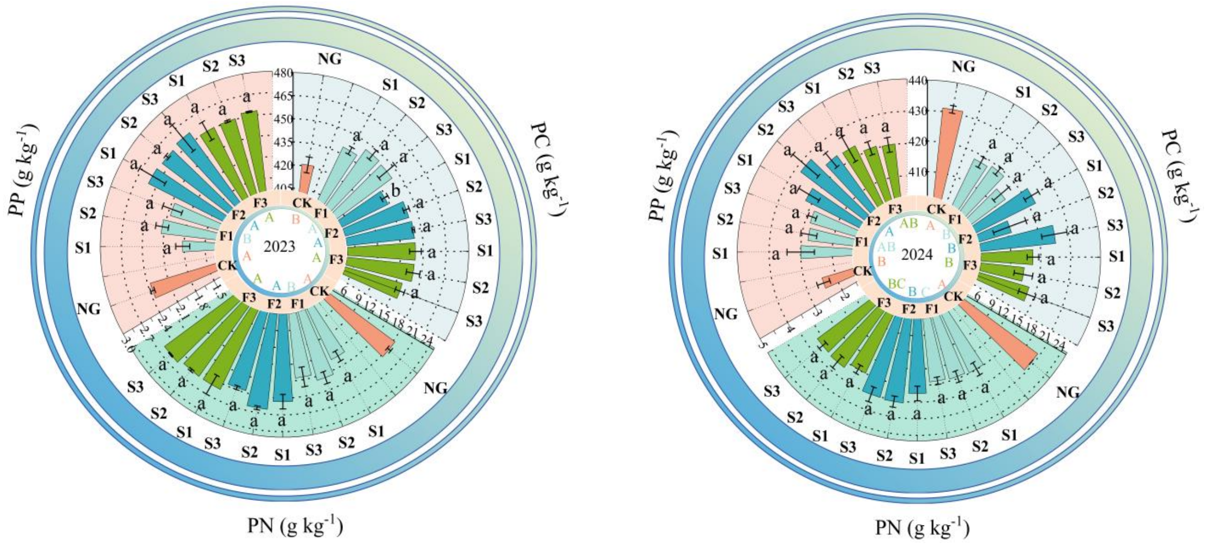

Figure S1. Effects of fertilizer application rate and seeding rate on plant carbon, nitrogen, and phosphorus contents.

Note: The lowercase letters in the figure indicate significant differences between seeding rates under the same fertilization treatment, while uppercase letters indicate significant differences between fertilization treatments and control. PC, PN and PP represent plant carbon, nitrogen and phosphorus nutrients, respectively.

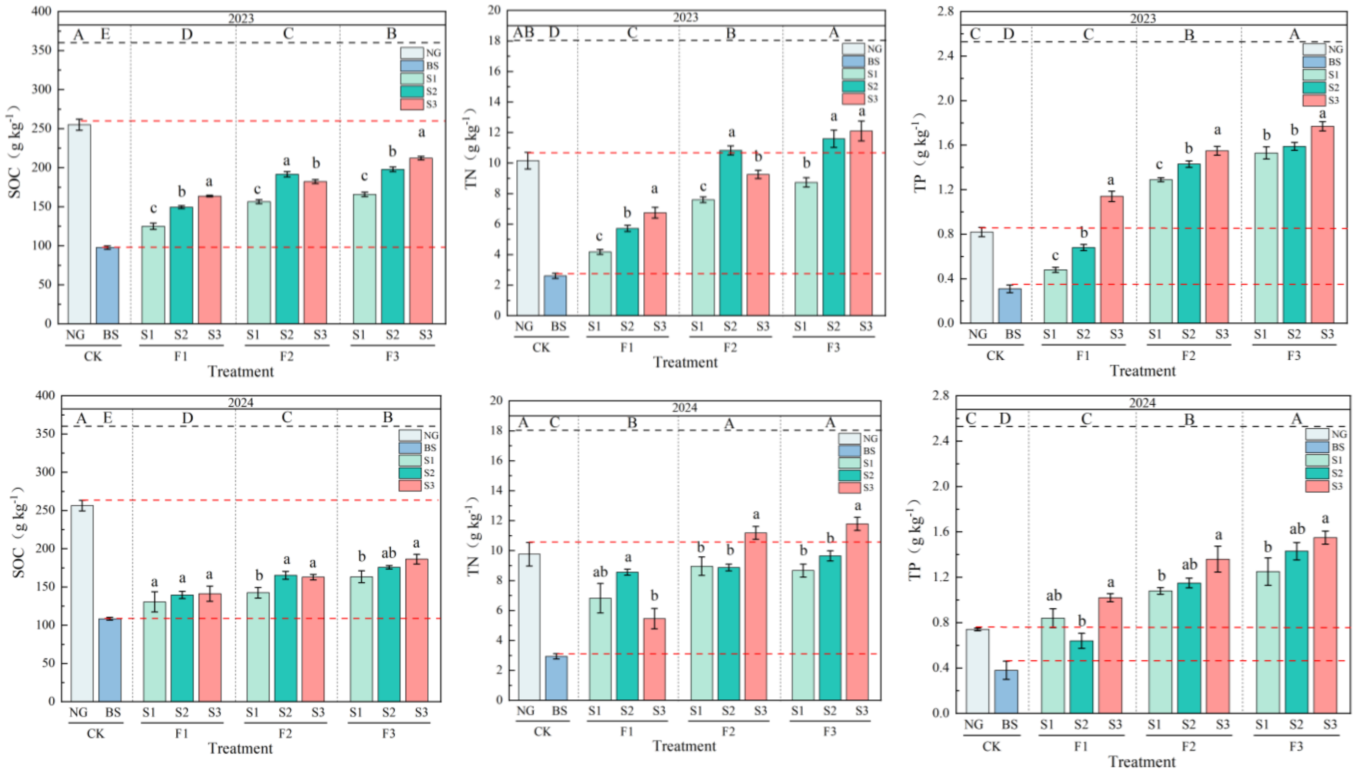

Figure S2. Effects of fertilization and seeding rate on soil carbon, nitrogen, and phosphorus contents.

Note: The lowercase letters in the figure indicate significant differences between seeding rates under the same fertilization treatment, while uppercase letters indicate significant differences between fertilization treatments and control. SOC, TN, TP respectively represent soil organic carbon, total nitrogen, total phosphorus content.

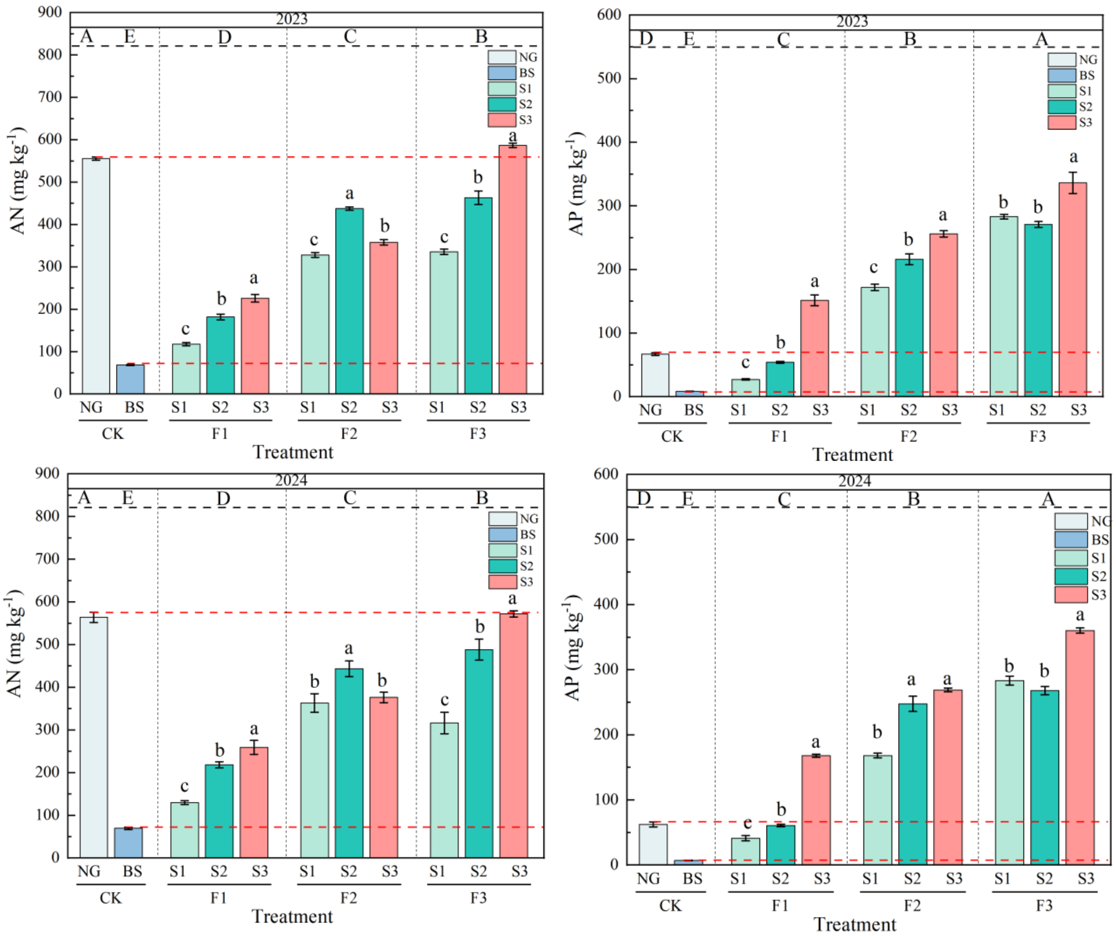

Figure S3. Effects of Fertilizer and Seeding Rates on Soil Available Nutrients

Note: The lowercase letters in the figure indicate that the difference between different seeding rates under the same fertilization treatment is significant, and Capital letter indicates that the difference between different fertilization treatments and the control is significant. AN and AP represent available nitrogen and available phosphorus, respectively.

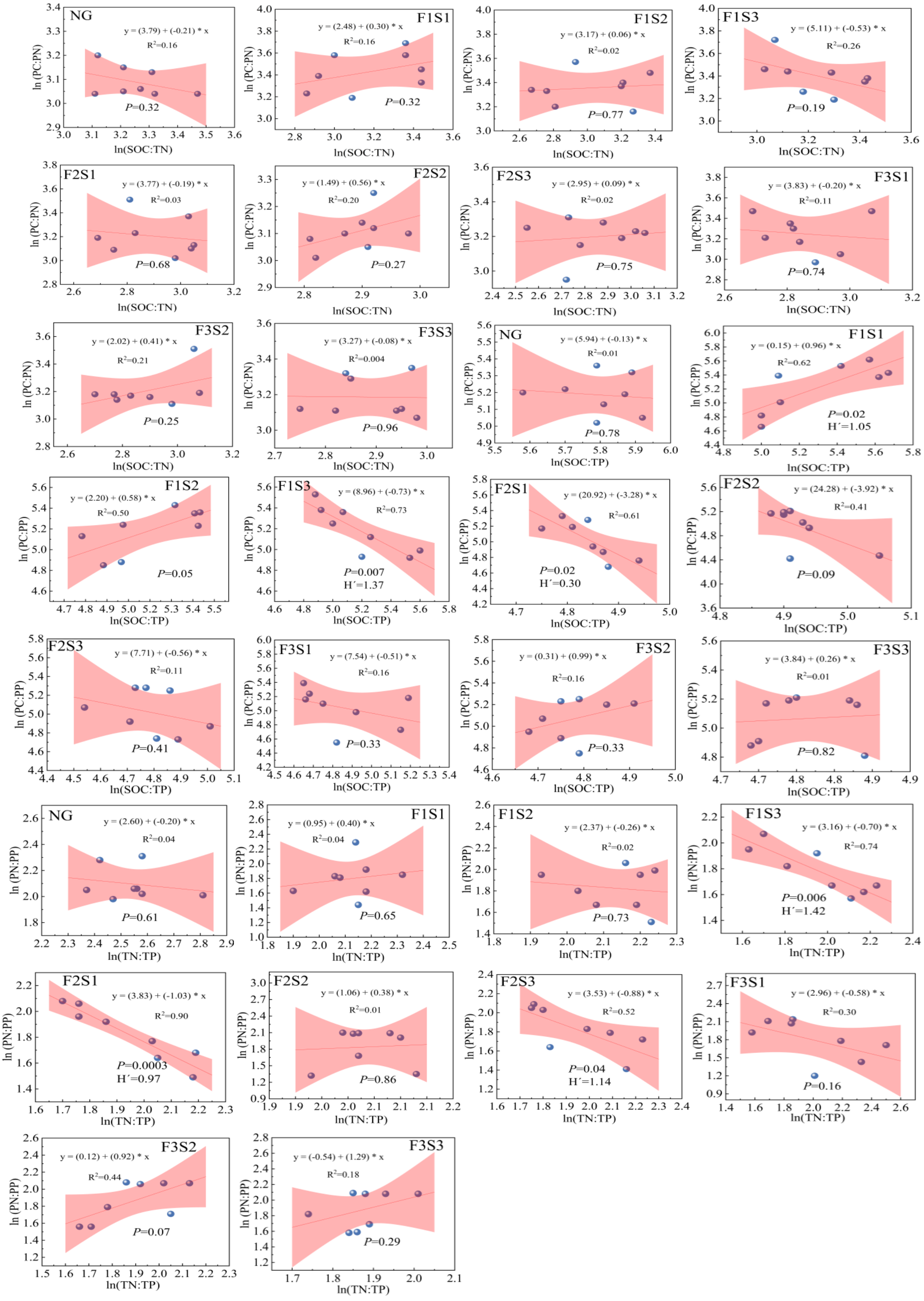

Figure S4. Analysis of the plant-soil stoichiometric homeostasis index.
